# Personalized cancer avatars for patients with thymic malignancies: A pilot study with circulating tumor cell‐derived organoids

**DOI:** 10.1111/1759-7714.15039

**Published:** 2023-07-20

**Authors:** Yuan‐Hung Wu, Heng‐sheng Chao, Chi‐Lu Chiang, Yung‐Hung Luo, Chao‐Hua Chiu, Sang‐Hue Yen, Chun‐Yu Liu, Jeng‐Fong Chiou, Thierry Burnouf, Yin‐Ju Chen, Peng‐Yuan Wang, Tsu‐Yi Chao, Shih‐Ming Hsu, Long‐Sheng Lu

**Affiliations:** ^1^ Department of Oncology Taipei Veterans General Hospital Taipei Taiwan; ^2^ School of Medicine National Yang‐Ming Chiao‐Tung University Taipei Taiwan; ^3^ Department of Biomedical Imaging and Radiological Sciences National Yang‐Ming Chiao‐Tung University Taipei Taiwan; ^4^ Department of Chest Medicine Taipei Veterans General Hospital Taipei Taiwan; ^5^ Taipei Cancer Center and Taipei Medical University Hospital Taipei Medical University Taipei Taiwan; ^6^ Department of Radiation Oncology Taipei Municipal Wan‐Fang Hospital Taipei Taiwan; ^7^ Department of Radiology, School of Medicine, College of Medicine Taipei Medical University Taipei Taiwan; ^8^ Department of Radiation Oncology Taipei Medical University Taipei Taiwan; ^9^ TMU Research Center of Cancer Translational Medicine Taipei Medical University Taipei Taiwan; ^10^ Graduate Institute of Biomedical Materials and Tissue Engineering, College of Biomedical Engineering Taipei Medical University Taipei Taiwan; ^11^ International Ph.D. Program for Cell Therapy and Regenerative Medicine, College of Medicine Taipei Medical University Taipei Taiwan; ^12^ Department of Radiation Oncology Taipei Medical University Hospital Taipei Taiwan; ^13^ Department of Medical Research Taipei Medical University Taipei Taiwan; ^14^ Oujiang Laboratory Wenzhou China; ^15^ Key Laboratory of Alzheimer's Disease of Zhejiang Province, Institute of Aging Wenzhou Medical University Wenzhou China; ^16^ Graduate Institute of Clinical Medicine, College of Medicine Taipei Medical University Taipei Taiwan; ^17^ Division of Hematology/Oncology, Department of Medicine, Tri‐service General Hospital National Defense Medical Center Taipei Taiwan; ^18^ Division of Hematology and Oncology, Department of Internal Medicine Taipei Medical University‐Shuang Ho Hospital New Taipei City Taiwan; ^19^ Taipei Cancer Center Taipei Medical University Taipei Taiwan

**Keywords:** circulating tumor cell, organoid, thymic carcinoma, thymic malignancy, thymoma

## Abstract

**Background:**

Systemic therapy is the primary treatment for advanced thymic malignancies. However, there is an urgent need to improve clinical outcome. Personalized treatment based on predictive biomarkers is a potential approach to address this requirement. In this study, we aimed to show the correlation between drug sensitivity tests on CTCs‐derived organoids and clinical response in patients with thymic malignancies. This approach carries the potential to create personalized cancer avatars and improve treatment outcome for patients.

**Methods:**

We previously reported potential treatment outcome prediction with patient‐derived organoids (cancer avatars) in patients with pancreatic ductal adenocarcinoma. To further investigate the feasibility of this approach in advanced thymic malignancies, we conducted a study in which 12 patients were enrolled and 21 liquid biopsies were performed.

**Results:**

Cancer avatars were successfully derived in 16 out of 21 samples (success rate 76.2%). We found a sensitivity of 1.0 and specificity of 0.6 for drug sensitivity tests on the cancer avatars, and a two‐tailed Fisher's exact test revealed a significant correlation between drug sensitivity tests and clinical responses (*p* = 0.0275).

**Conclusion:**

This study supports the potential of circulating tumor cell‐derived organoids to inform personalized treatment for advanced thymic malignancies. Further validation of this proof of concept finding is ongoing.

## Graphical abstract

Cancer avatars were successfully derived in 16 out of 21 samples (success rate 76.2%) of patients of advanced thymic malignancies. We found a sensitivity of 1.0 and specificity of 0.6 for drug sensitivity tests on the cancer avatars, and a two‐tailed Fisher's exact test revealed a significant correlation between drug sensitivity tests and clinical responses (*p* = 0.0275).

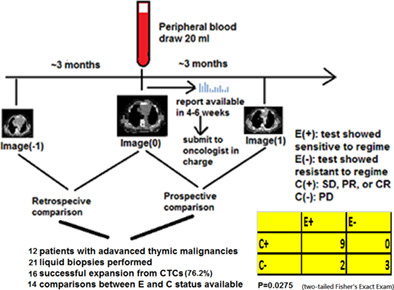

## INTRODUCTION

Thymic malignancies are rare types of cancer that occur in the mediastinum, with thymoma and thymic carcinoma being the most common types. These cancers account for 0.2%–1.5% of all malignancies in the United States.^1^ Surgery is the primary treatment option for early‐stage thymic malignancies, but systemic therapy is typically used for advanced or recurrent disease. Currently, the choice of systemic treatment relies on empirical regimes. According to NCCN guidelines for thymoma and thymic carcinoma, the recommended frontline regimens for thymoma are CAP (cisplatin, doxorubicin, and cyclophosphamide) and carboplatin/paclitaxel for thymic carcinoma.^2^, ^3^, ^4^ The objective response rate of CAP on thymoma is around 50%, while only 21.7% with carboplatin/paclitaxel for thymic carcinoma. This indicates a significant unmet need to improve the treatment outcome. However, the development of new therapies is limited by the disease rarity as well as the elusive nature of its driving pathogenic mechanisms. Therefore, an alternative plan is needed.

Recently, significant technological improvement has enabled the possibility of personalized cancer medicine. A promising approach is to obtain cancer initiating cells from patient tumor samples. Drug sensitivity of these ex vivo cancer cells (cancer avatars) has been shown to predict personalized clinical treatment response in colorectal, prostate and breast cancers.^5^, ^6^, ^7^ For example, drug sensitivity towards regorafenib of organoids from surgical biopsy of metastatic colon cancer tissues have been shown to predict the clinical treatment response to regorafenib.^5^ Such technologies have been applied in thymus biology research, including studies of T cell development with artificial thymic organoids and therapeutic T cell engineering.^8^, ^9^, ^10^ Moreover, patient‐derived thymic tumor tissue has been analyzed in a similar approach to uncover the novel role of WNT4 in thymic epithelial carcinogenesis.^11^ While there is still a long development process for new biologically directed therapies for thymic malignancy, we hypothesize that drug sensitivity profile of tumor organoids may inform treatment decision by optimizing regimen selection.

A unique source of tumor organoids is circulating tumor cells (CTCs). CTCs are cancer cells found in the systemic circulation that originally extravasate microvascular barrier from a solid tumor. These cells are enriched in cancer initiating cells that carry stem cell characteristics and that mechanistically contribute to metastasis and disease progression. Elevated CTCs count are poor prognostic predictors in human malignancy including breast, colon and prostate cancers.^12^, ^13^ However, whether CTCs analysis can inform clinical disease progress or management for thymic malignancy is unknown. Our group recently developed a system for efficient tumor organoid derivation from CTCs. Clinical correlation studies in soft tissue sarcoma, pancreatic ductal adenocarcinoma and small lung cancer have demonstrated a correlation between drug sensitivity profiles and clinical response with this technology.^14^, ^15^ In this study, we aimed to show the correlation between drug sensitivity tests on CTCs‐derived organoids and clinical response in patients with thymic malignancies. This approach carries potential to create personalized cancer avatars and improve treatment outcomes for these patients.

## METHODS

### Patients

**FIGURE 1:**
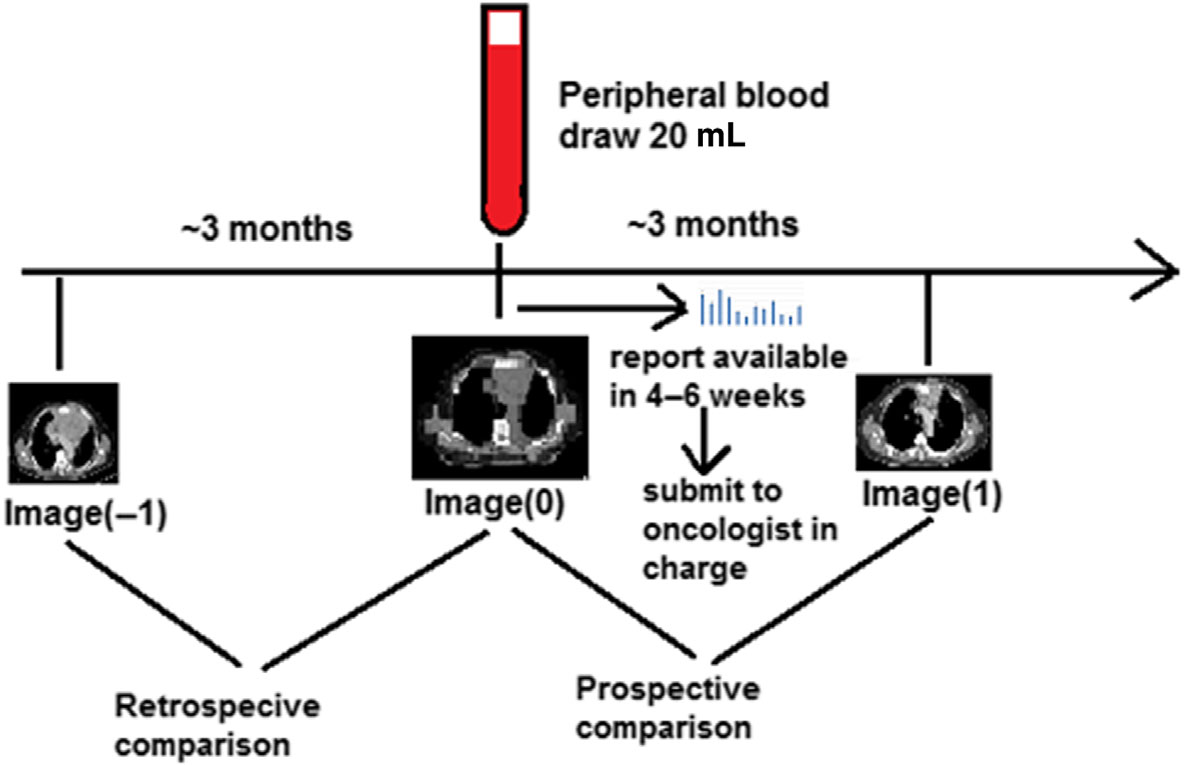
Flow chart of experimental procedures conducted for enrolled patients.

This observational study was approved by the institutional review board (IRB) of Taipei Medical University Hospital (protocol record N201803020) and retrospectively registered in ClinicalTrials.gov (NCT04972461). The study was conducted in accordance with institutional and local regulations, Good Clinical Practice (GCP), and the Declaration of Helsinki, and written informed consent was obtained from all patients prior to enrollment. From August 2018 to January 2023, 12 eligible patients with pathologically proved thymic malignancies were enrolled in the study. Inclusion criteria were: (1) age ≥20, (2) Karnofsky performance score (KPS) ≥70, (3) at least one measurable lesion, (4) life expectancy of >3 months, and (5) has not been treated with immune checkpoint inhibitors. Tumor staging was performed according to the American Joint Committee on Cancer (AJCC) Cancer Staging Manual (eighth edition) and clinical response was evaluated using Response Evaluation Criteria in Solid Tumors (RECIST) version 1.1 comparing medical images at the time of blood sampling and medical images performed at 3 ± 1 months before (retrospective comparison), and at 3 ± 1 months later on (prospective comparison) as shown in Figure 1.^16^, ^17^

### CTC extraction and organoid expansion

Liquid biopsies were performed after a minimum of 2 weeks following the completion of chemotherapy cycles. A total of 20 mL liquid biopsies of peripheral venous blood were collected from each patient using K2EDTA vacutainers (BD Bioscience), with 19 liquid biopsies collected in total, from which the peripheral blood mononuclear cell (PBMC) fraction, which contains CTCs, was isolated through Ficoll‐Paque centrifugation as previously described.^14^, ^15^, ^18^ Subsequently, the CTCs were enriched using a RosetteSep CTC Enrichment Cocktail kit (Stem Cell Technologies). The E.V.A. Select system employed in this process does not rely on size elimination or epithelial marker capture methods. This approach ensures the preservation of CTCs heterogeneity, resulting in organoid cultures that better represent the actual conditions of the tumor.

The cells were then seeded onto a binary colloidal crystal (BCC) substrate containing silica and polymethyl methacrylate (PMMA). A platelet lysate‐based culture medium (DMEM/F12 medium with platelet lysate and B27 supplement; Thermo Fisher Scientific Inc.) supplemented with 0.01% epidermal growth factor (EGF) (Thermo Fisher Scientific Inc.), 0.01% basic fibroblast growth factor (bFGF) (Thermo Fisher Scientific Inc.), and 10% platelet‐derived growth factor was used as a complete culture medium for whole process culturing.^19^ This process took place in the incubator at a temperature of 37°C with 5% CO_2_ and 95% humidity for a duration of 3 weeks. The complete culture medium was replaced every 4 days. It is important to note that this two‐dimensional culture method did not involve the use of an extracellular matrix. The system represents a novel approach developed from a previously published stem cell culture method.^20^

Organoid expansion was monitored by optic microscopy. CTCs presence after the culture is defined by the presence of cell clusters that stains positive for Pan‐cytokeratin and negative for CD45. We quantified samples for drug testing by measuring adenosine triphosphate (ATP) through bioluminescence, which indicates the presence of live cells. ATP abundance was expressed as relative light units (RLUs). Organoids were divided into 2000 RLUs aliquots for drug testing, ensuring sufficient viable cells for assessing drug sensitivity. Samples that did not successfully expand during the CTC expansion process were labeled as “failed expansion”. In this research, CTCs counting in the initial sample was not performed. One‐eighth of CTCs were sampled for immunofluorescence analysis, and the remaining CTCs were submitted to drug sensitivity tests. In the lab, we estimate cell count with ATP abundance. Cells have been submitted to a drug sensitivity test if ATP abundance is higher than 2500 units, which corresponds to a cell count of 12 000 with the reference cell line A549. In the literature of metastatic breast cancer research, in most samples, the number of CTCs ranges between 1 and 100 per 7.5 mL blood.^21^ Therefore, even with the low estimate of 12 000 cells, CTCs were expanded by at least two orders in our system.

### Drug sensitivity profiling

Circulating tumor cell‐derived organoids were resuspended in  the culture medium and loaded into 96‐well plates for drug sensitivity assays at clinically relevant concentrations. Specifically, we utilized a single concentration derived from the maximum plasma concentration (*C*_max_) as calculated in the referenced paper.^22^ Tests were conducted in triplicate for each drug, and cultures were continued for 1 week after drug treatment. The abundance of cytosolic ATP was then measured by luminometer (CellTiter Glo, Promega) to determine viable cell counts, which were normalized to untreated control groups to derive relative cell viability (RCV). From previous experience, we set the threshold of RCV as 15% for gemcitabine and 50% for the rest drugs. The binary drug sensitivity variable (E) was assessed for each liquid biopsy and defined as positive (E+) if patients received treatment with any drug other than gemcitabine that had a relative cell viability (RCV) <50%, or gemcitabine with RCV <15%. Patients with treatments except for gemcitabine that all had an RCV ≥50% and with RCV of gemcitabine ≥15% were defined as negative (E−). Patient treatments were designated as “unmatched” if there were no corresponding drug sensitivity results available, or the target lesion received radiotherapy within 3 months. Patients with failed expansion, received no medical treatment, or with no clinical response available were assigned an E status of N/A (not applicable).

### Clinical response assessment

Medical images from patients taken 3 ± 1 months prior to (Image(−1)), within 1 month (Image(0)), and 3 ± 1 months after (Image(1)) liquid biopsies (Figure 1) were independently assessed for clinical response by two radiation oncologists blinded to patient medications and drug sensitivity results, and scored as complete response (CR), partial response (PR), stable disease (SD), or progressive disease (PD).^16^

Circulating tumor cells were isolated and expanded ex vivo from patient liquid biopsies to form CTC‐derived organoid cultures, which were then tested for sensitivity to specific drugs. The report of drug sensitivity tests, usually available 4–6 weeks after liquid biopsy, would be submitted to the oncologist in charge if the expansions and tests were successful. The oncologist may or may not change the regime, depend on individual discretion. Drug sensitivity results were compared with clinical response to treatment(s) received for a minimum of 6 weeks in the 3‐month period before or after liquid biopsy (retrospective/ prospective comparisons).

### Correlating drug sensitivity profiles with clinical response

Drugs received by patients for a duration of more than 6 weeks in the 3 months after liquid biopsy were included for retrospective and prospective comparisons between CTC‐derived organoid drug sensitivity (E) and clinical response (CR/PR/SD/PD; Figure 1), with E status (E+ vs. E−) compared against a binary clinical benefit variable C (C+ vs. C−), with C+ defined as CR, PR, or SD, and C− defined as PD.

### Statistical analysis

To test the correlation between E‐status and C‐status, we utilized a two‐tailed Fisher's exact test on the 2 by 2 contingency table. Statistical significance was determined by a *p*‐value < 0.05. For the inferential statistics, we employed the online tool available on graphpad.com.

## RESULTS

### Patient demographics

**FIGURE 2:**
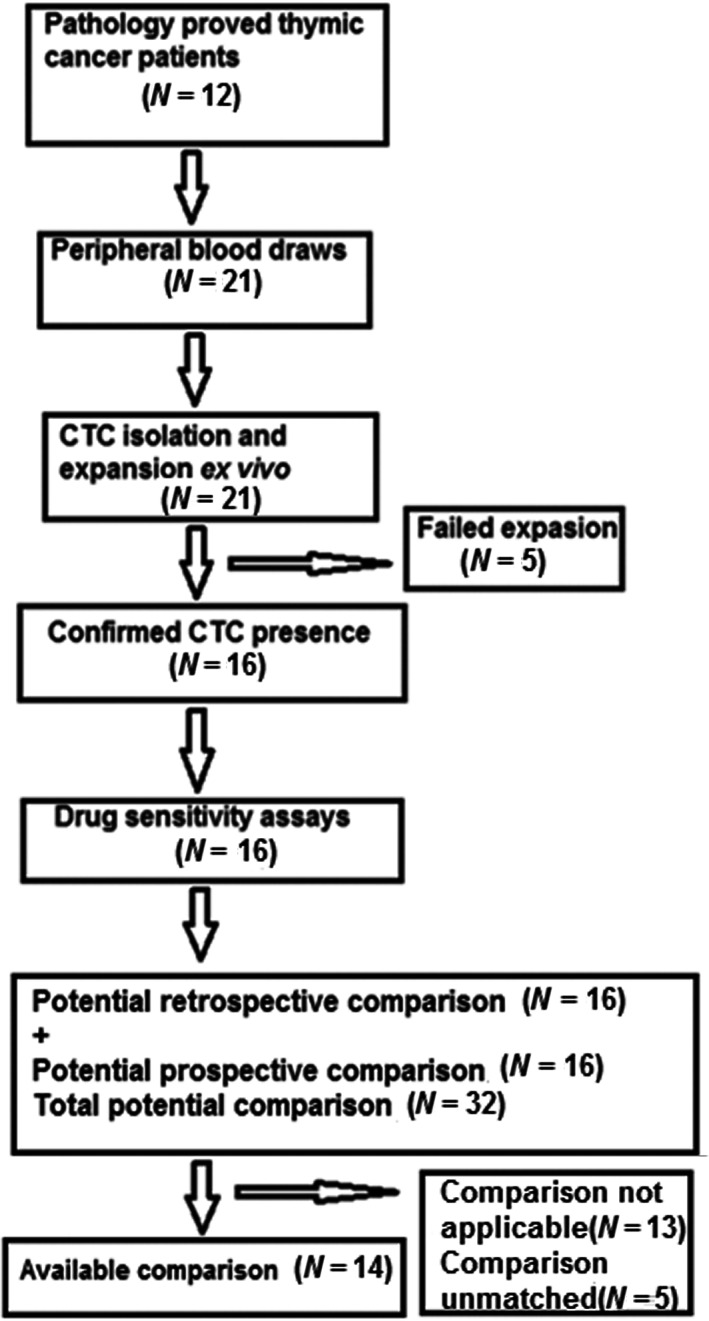
CTC‐derived organoids expanded ex vivo from thymic malignancy patient CTCs.

Patient demographics of the 12 enrolled patients are presented in Table 1, and the study flow is shown in Figure 2. Female patients made up 58.3%, and mean age was 47.2 years, with the eldest patient being 61 years old. Most (83.3%) patients had stage 4 disease. CTCs expansion failed for five liquid biopsies. Results from 16 liquid biopsies were included in retrospective/prospective comparisons.

**TABLE 1:** Patient demographics of all 12 enrolled patients.

Mean age, years	47.2 (26–61)
Female, *n* (%)	7 (58.3%)
Cancer staging at 1st time of liquid biopsy	
Stage 4, *n* (%)	10 (83.3%)
Stage 3, *n* (%)	2 (16.7%)
Pathology	
Squamous cell carcinoma, *n* (%)	5 (41.7%)
Thymoma, *n* (%)	3 (25%)
Poorly differentiated carcinoma, *n* (%)	1 (8.3%)
Poorly differentiated non‐small cell carcinoma in combination with a small cell carcinoma, *n* (%)	1 (8.3%)
Mucinous adenocarcinoma, *n* (%)	1 (8.3%)
Adenocarcinoma, *n* (%)	1 (8.3%)

### CTC expansion

**FIGURE 3:**
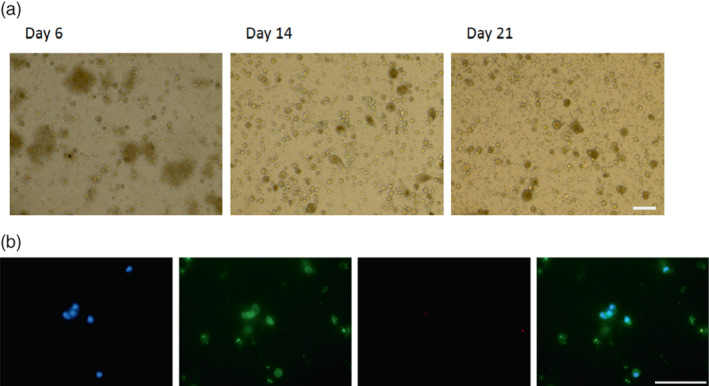
CTC‐derived organoids expanded ex vivo from the illustrative case (patient no. 5 in the Table S1) with thymic squamous cell carcinoma CTCs. (a) bright field images of CTCs‐derived organoids. Scale bar: 100 μm. (b) DAPI, pan‐cytokeratin, CD45, and merge staining results from the illustrative case to confirm the presence of CTCs. Scale bar: 75 μm.

Circulating tumor cells were obtained from 21 fresh liquid biopsies collected from 12 patients. At the beginning of the experiment (Day 0), peripheral blood mononuclear cells (PBMCs) that contained enriched CTCs were placed in three‐dimensional 24‐well plates. Between Days 7 and 28, the cells changed shape and grew in size, eventually forming organoids (Figure 3a). After 3 weeks of culturing, CTCs were confirmed by immunofluorescence staining for Pan‐cytokeratin (a protein expressed by CTCs) and the absence of CD45 (a protein expressed by leukocyte cells) (Figure 3b). This process was successful in cultivating CTCs‐derived organoids from 76.2% (16/21) of the biopsies. The organoids were then suspended in the culture medium and placed in 96‐well plates for drug sensitivity testing.

### Correlation between drug sensitivity tests and clinical responses

Combining retrospective and prospective comparisons of drug sensitivity variable E status and the clinical response C status as denoted in Table 2. Sensitivity of the cancer avatar, as defined by (C+E+)/[(C+E+) + (C+E−)], is 9/9 = 1. Specificity, as defined by (C−E−)/[(C−E+) + (C−E−)], is 3/(2 + 3) = 0.6. Two‐tailed Fisher's exact test on the 2 by 2 contingency table (Table 2) found a significant *p*‐value of 0.0275.

**TABLE 2:** Correlation table on drug sensitivity tests on cancer avatars (E‐status) and clinical responses of the patients (C‐status).

	E+	E−
C+	9	0
C−	2	3

### Illustrative cases

#### Personalized chemotherapy after exhausting standard treatment options

**FIGURE 4:**
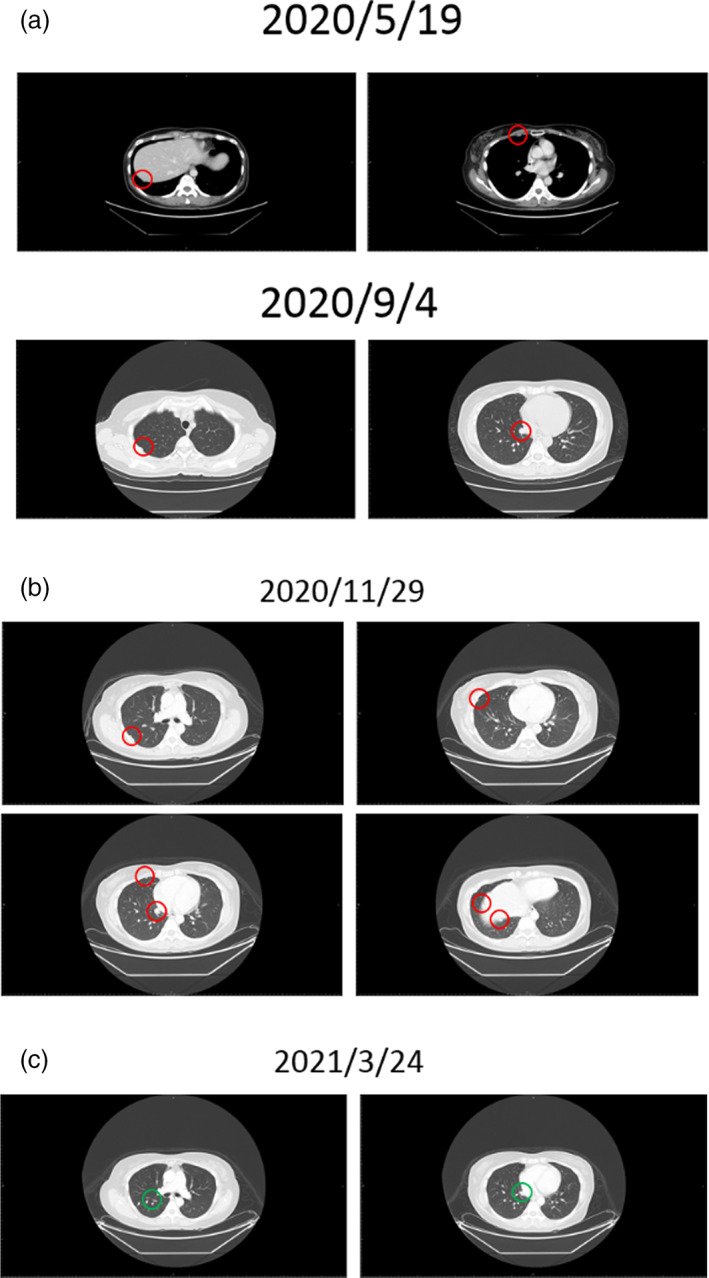
Serial CT scan showed clinical responses of an illustrative case (patient #5).

A 39‐year‐old woman with a family history of thymic and colorectal cancer was diagnosed with thymic squamous cell carcinoma, pT3N1M0, stage III in March 2018. She received surgery, postoperative radiotherapy and chemotherapy with cisplatin. The disease recurred in May 2020 as masses in the right chest wall and right lower lung abutting diaphragm were found on a computed tomography (CT) scan. Despite complete resection of the oligo‐metastatic tumors, further progression was found 3 months later as two more nodules in the right lower lung (Figure 4a). A surgical specimen of the resected lung nodules from the most recent surgery was sent for multigene panel profiling and her blood sample was sent for CTCs‐derived organoid drug sensitivity profiles. Gene profiling revealed pathogenic alterations including TP53 point mutation (G245S), and frameshift mutations of CDKN2A/B and MLL2. CTC organoid drug sensitivity revealed resistance to platinum and potential sensitivity to doxorubicin, 5‐fluorouracil, gemcitabine and eribulin (Table 3). She received chemotherapy with VIP regimen (cisplatin, etoposide and ifosfamide) but additional tumor seedings were found 2 months later (Figure 4b). Then she shifted to doxorubicin containing regimen with cisplatin, doxorubicin, and cyclophosphamide since November 2020. This time a partial response was documented 3 months later (Figure 4c). Then she started eribulin and disease remained controlled until February 2022. Throughout the treatment course, there were no grade 2 or higher side effects. This case illustrates how CTCs‐derived organoids as cancer avatars may aid treatment decision when standard treatment options were exhausted and gene panel profiling provided limited actionable options.

**TABLE 3:** Drug sensitivity tests on cancer avatars of the illustrative case (patient #5).

Drug	Concentration (μM)	Viability (%)	
Liquid biopsy sampled on September 24, 2020			
Carboplatin	13.5	71	
Cisplatin	1.44	89	
Doxorubicin	0.673	44	
Paclitaxel	4.27	66	
Pemetrexed	30.6	71	
Sorafenib	20.1	56	
Sunitinib	0.181	71	
Liquid biopsy sampled on January 21, 2021			
Palbociclib	0.101	56
Eribulin	0.508	15
Olaparib	13.1	71
Fluorouracil	30	34
Irinotecan	5.78	44
Gemcitabine	10	8.3

#### Treatment failure to sorafenib retrospectively revealed by cancer avatars

**FIGURE 5:**
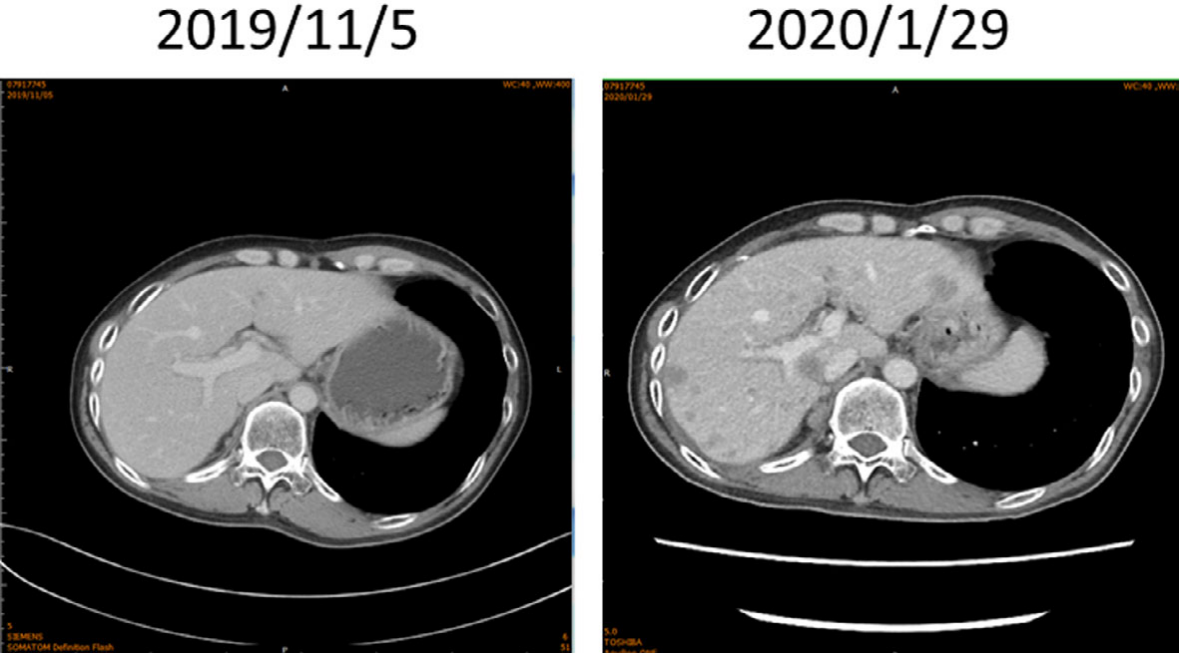
Serial CT scans showed liver metastasis progression during sorafenib treatment.

A 37‐year‐old man without family cancer history or predisposing risk factor was diagnosed with thymic carcinoma in December 2009. Initially he received neoadjuvant chemotherapy followed by radical thymectomy and postoperative radiotherapy. The tumor relapsed as a single metastatic lung mass in right lower lobe 9 months later. Despite thoracoscopic resection, the disease progressed to the right middle lobe with seeding to adjacent pleura and diaphragm in May 2013. Following resection of lesions in the right middle lobe, diaphragm and partial pleurectomy, he received chemotherapy with high dose 5‐fluorouracil and leucovorin. Further disease progression to bilateral pleural space, mediastinum and peritoneum was found 4 months later. However, the disease responded to compassionate adoptive dendritic cell therapy concurrent with palliative radiotherapy. The disease remained stable for the following 6 years without further chemotherapy. In December 2019, he experienced mild fatigue and CT scan revealed limited metastases in the liver. The patient opted for sorafenib and rapid progression in the liver was found in January 2021 (Figure 5). CTC organoid drug sensitivity later in February 2020 revealed resistance to sorafenib, which corresponded to the clinical response (Table 4). He was treated with palliative doxorubicin and the disease was stabilized for 6 months. Unfortunately, pleural metastases with pulmonary artery thrombosis ensued and he finally succumbed to respiratory failure in August 2021.

**TABLE 4:** Drug sensitivity tests on cancer avatars of the illustrative case (patient no. 14).

Liquid biopsy sampled on February 3, 2020		
Drug	Concentration (μM)	Viability (%)
Carboplatin	13.5	100
Docetaxel	5.47	28
Enzalutamide	35.7	52
Etoposide	33.4	100
Everolimus	0.064	17
Mitomycin C	0.218	24
Paclitaxel	4.27	66
Palbociclib	0.101	80
Pemetrexed	30.6	100
Sorafenib	20.1	65
Vinorelbine	0.811	76

## DISCUSSION

In this study, a biomimetic cell culture system was applied to derive CTCs organoids from peripheral blood of patients with thymic malignancies. The system had a culture success rate of 73.7% and it took less than 4 weeks for drug sensitivity analysis. The workflow has the potential to fit in the clinical setting of personalized cancer therapy after palliative radiotherapy. When these CTCs‐derived organoid served as personalized cancer avatars, the results of drug sensitivity test correlated with clinical responses in thymic malignancies patients. While it reproduced the findings in pancreatic ductal adenocarcinoma and small cell lung cancer, this is the first study to show a potential cell‐based predictive biomarker for thymic cancer. Deriving cancer organoids from CTCs in peripheral blood is a less invasive alternative comparing to doing so from a surgical specimen. Moreover, sampling cancer initiating cells from CTCs is less likely to be biased by spatial tumor heterogeneity that is a common pitfall for sampling these elements from a surgical biopsy. Personalized cancer avatars with the E.V.A. Select system or equivalents have the potential to screen clinically actionable cytotoxic/cytostatic options of anti‐cancer drugs and combinations in several different situations. For example, it could be used to find out the most and the least cytotoxic drug in the specific context at the outset of disease progression. It could also be used to monitor the effectiveness of a particular treatment over time, including after the development of resistance or relapse. This information may play a role to inform a treatment plan at patient‐specific resolution.

Treatment for refractory thymic cancer is a clinical challenge. Currently the standard chemotherapy remains empirical. Response rate from platinum with anthracycline‐based chemotherapy was less than 70% for thymoma and around 40% for thymic carcinoma.^23^ To improve, a clearer understanding of biology in thymic epithelial tumors is needed. It has been pointed out that thymic epithelial tumors depend on tumor angiogenesis and growth signals from c‐KIT and EGFR for propagation.^24^ Genomic landscape analyses from The Cancer Genome Atlas (TCGA) collection revealed enrichment of HRAS, NRAS, TP53 GTF2I mutations and chromosome 16q loss.^25^ Comprehensive genomic profiling of recurrent, refractory 90 thymoma and 174 thymic carcinoma patients showed a low frequency of genomic alterations (average of 1.8/tumor) and low levels of tumor mutation burden (TMB) for thymoma. Amplification in the NTRK1 gene was found in a patient with thymoma. Thymic carcinoma featured a higher frequency of alterations at 4.0/tumor. Clinically relevant genomic alterations were observed in the CDKN2A, KIT, and PTEN/PI3K/MTOR pathways. Elevated TMB in thymic carcinoma was uncommon with only 6% of cases featuring ≥10 mutations/Mb.^26^ Biologically targeted therapies are developing. Small molecule inhibitors of angiogenesis including regorafenib, sunitinib, and lenvatinib showed response rates ranging from 0% to 38% in patients with advanced thymic carcinoma The following emerging drug targets are under evaluation in thymic epithelial tumors: the mediator of nucleus‐cytoplasmic shuttling XPO1; the PI3K/mTOR pathway; the CDK/Rb pathway; NTRK 1/2/3, and the MTAP enzyme.^27^ Targeted therapy with imatinib guided by NGS was reported to treat a patient with metastatic thymic squamous cell carcinoma.^28^ Cell‐guided therapy using ex vivo drug screen on tumor derived primary cell culture has also been reported in a patient with recurrent thymoma.^29^ To the best of our knowledge, the current study is the first to report correlation between drug sensitivity tests on CTC‐derived organoid and clinical response for patients with thymic malignancies. From the promising result of the current study, CTC‐guided therapy may be feasible.

This study had several limitations. First, the retrospective, observational study design introduces heterogeneity in treatment and follow‐up. Second, the rarity of patients with metastatic thymic malignancy and the low response rate of palliative systemic therapy limits the sample size included in the investigation. Last. but not the least, the E.V.A. select system used in this study did not incorporate tumor microenvironment components, and therefore the impact of drugs affecting angiogenesis and the immune system could not be assessed. While the pilot study showed statistical significance in a small patient cohort, validation of the finding with a powered, controlled trial is necessary before further routine clinical application.

In conclusion, we found drug sensitivity tests on cancer avatars derived from CTCs of peripheral blood of patients with thymic malignancies may be promising in correlating to the clinical responses.

## AUTHOR CONTRIBUTIONS

Conceptualization, YHW and LSL; methodology, TB, YJC, PYW, and LSL; validation, SHY, CHC, YHL, HSC, CLC, CYL, TYC, LSL; formal analysis, SHY; investigation, YHW; resources, JFC and LSL; data curation, YHW; writing—original draft preparation, YHW; writing—review and editing, YHW, LSL; visualization, YHW; supervision, SMH and LSL; project administration, LSL; funding acquisition, JFC and LSL. All authors have read and agreed to the published version of the manuscript.

## FUNDING INFORMATION

This work was supported by the TMU Research Center of Cancer Translational Medicine from the Featured Areas Research Center Program within the framework of the Higher Education Sprout Project by the Ministry of Education in Taiwan (grant numbers DP2‐110‐21121‐03‐C‐06‐01, DP2‐110‐21121‐03‐C‐06‐02, DP2‐110‐21121‐03‐C‐06‐03, DP2‐109‐21121‐03‐C‐06‐01, DP2‐109‐21121‐03‐C‐06‐02, DP2‐109‐21121‐03‐C‐06‐03); the Ministry of Science and Technology (grant nos. MOST‐111‐2923‐B‐038‐001‐MY3, MOST‐111‐2314‐B‐038‐070‐MY3, MOST‐110‐2320‐B‐038‐056, MOST‐110‐2314‐B‐038‐138, MOST‐109‐2314‐B‐038‐122, MOST‐109‐2314‐B‐038‐141, MOST‐109‐2635‐B‐038‐001, MOST‐109‐2314‐B‐038‐072); Taipei Medical University (grant no. TMU105‐AE1‐B13); and National Health Research Institutes (grant no. NHRI‐EX109‐10713EI). The funders had no role in the design of the study, collection, analysis, and interpretation of the data, or the writing and publication of this manuscript.

## CONFLICT OF INTEREST STATEMENT

Long‐Sheng Lu, Yin‐Ju Chen, Peng‐Yuan Wang, and Thierry Burnouf are listed as inventors in the patent for E.V.A. Select., Yuan‐Hung Wu holds stock shares in CancerFree Biotech Ltd, a company that has obtained a license for the E.V.A. Select technology from Taipei Medical University. The remaining authors state that they have no conflicts of interest to disclose.

## CONSENT

All patients provided their written informed consent prior to enrollment.

## Supporting information


Table S1.

## Data Availability

Detailed patient data, cancer medications, and test results of each liquid biopsy are provided in the supplement. The other data generated in this study were not made publicly available due to patient privacy and ethical considerations. However, upon reasonable request, the data may be made available from the corresponding author which is subject to approval by the IRB of Taipei Medical University Hospital.
